# Neurosurgical applications of MRI guided laser interstitial thermal therapy (LITT)

**DOI:** 10.1186/s40644-019-0250-4

**Published:** 2019-10-15

**Authors:** Usama Salem, Vinodh A. Kumar, John E. Madewell, Donald F. Schomer, Dhiego Chaves de Almeida Bastos, Pascal O. Zinn, Jeffrey S. Weinberg, Ganesh Rao, Sujit S. Prabhu, Rivka R. Colen

**Affiliations:** 1 Department of Radiology, The University of Texas Medical Branch at Galveston, Galveston, TX 77555 USA; 2 Department of Radiology, The University of Texas MD Anderson Cancer Center, Houston, TX 77030 USA; 3 Department of Neurosurgery, The University of Texas MD Anderson Cancer Center, Houston, TX 77030 USA; 4 Department of Radiology, University of Pittsburgh Medical Center, Pittsburgh, PA 15232 USA; 5 Hillman Cancer Center, University of Pittsburgh Medical Center, Pittsburgh, PA 15232 USA

**Keywords:** Laser interstitial thermal therapy, Laser ablation

## Abstract

MRI-guided laser interstitial thermal therapy (LITT) is the selective ablation of a lesion or a tissue using heat emitted from a laser device. LITT is considered a less invasive technique compared to open surgery that provides a nonsurgical solution for patients who cannot tolerate surgery. Although laser ablation has been used to treat brain lesions for decades, recent advances in MRI have improved lesion targeting and enabled real-time accurate monitoring of the thermal ablation process. These advances have led to a plethora of research involving the technique, safety, and potential applications of LITT.

LITT is a minimally invasive treatment modality that shows promising results and is associated with decreased morbidity. It has various applications, such as treatment of glioma, brain metastases, radiation necrosis, and epilepsy. It can provide a safer alternative treatment option for patients in whom the lesion is not accessible by surgery, who are not surgical candidates, or in whom other standard treatment options have failed. Our aim is to review the current literature on LITT and provide a descriptive review of the technique, imaging findings, and clinical applications for neurosurgery.

## Background

MRI-guided laser interstitial thermal therapy (LITT) is the selective ablation of a lesion or a structure using heat liberated from a laser [1]. LITT has been used for a variety of lesions in various organs such as lung, liver, bone, and prostate. In the brain, LITT is considered a less invasive procedure than open surgery, performed under real-time MRI guidance to treat several intracranial pathologies [2]. Although other techniques have been used for focal tissue ablation, such as radiofrequency, cryo-, and microwave ablation, laser ablation has superior precision and predictable volume of tissue ablation thus avoiding collateral damage [3]. In this review, we present background on LITT in neurosurgery and discuss the technique, indications, and potential complications of MRI-guided LITT.

### History of lasers in neurosurgery

The application of lasers in the brain started in 1965, when a pulsed ruby laser was used on the cranium of mice and guinea pigs, leading to immediate death [4]. The cause of death was sudden increased intracranial pressure due to the explosive interaction of the laser and the brain tissue in a closed cranium [5–7]. Later, the pulsed ruby laser was used on exposed brains of cats, leading to hemorrhagic lesions at the site of impact [8]. The aim of these early reports was to study the destructive effect of laser on tissues before human clinical trials. The first report of using a laser in a human was in 1966, when a pulsed ruby laser was selectively focused on a brain tumor, leading to incomplete tumor necrosis [9]. The partial effect was attributed to the poor absorption of the laser by the pigmented tissues, which made it difficult to control the thermal effect on neural tissue [9]. In addition, the photomechanical effects of the pulsed laser were uncontrolled [9]. This early report showed that the procedure was feasible, although further research was required to achieve proper and selective lesion targeting and hence adequate lesion ablation. To overcome the explosive interaction of the laser with tissue and to obtain more accurate and controlled laser energy, a continuous-wave laser with an improved delivery system was used [7]. In 1966, a CO_2_ laser, which is a high-power continuous-wave laser, was used to vaporize a recurrent glioma. Although the procedure was precise and controlled, it was a time-consuming procedure and consequently impractical [10]. During 1976–1979, Asher and Heppner [11–13] performed more than 250 central nervous system (CNS) lesion ablations after modifying the CO_2_ laser. They added a visible helium laser to guide the surgeon to precisely direct the invisible CO_2_ laser. In addition, they coupled the laser to an operating microscope to increase precision. The result was a powerful microsurgical scalpel that they used for extra-axial tumors and small intra-axial vascular lesions [11, 12].

A neodymium-doped yttrium aluminum garnet (Nd:YAG) laser was also used but lacked the precision required for most neurosurgical procedures owing to poor absorption by CNS tissue, leading to extensive collateral damage. Because the Nd:YAG laser is selectively absorbed by blood and blood vessels, it can be used to occlude small blood vessels [14, 15]. In addition, studies on rabbit brains showed that the Nd:YAG laser penetrated deeper than the CO_2_ laser; the depth of penetration was predictable, and its effect on vascularized tissues was greater than that of the CO_2_ laser [16].

In 1983, Bown used the Nd:YAG laser to induce focal tissue coagulation in an experimental brain model, which led to the development of LITT [17, 18]. After several clinical trials, in 1990, Sugiyama et al. reported the clinical application of LITT to treat 5 patients with brain tumors [19]; the ablation procedure was performed under CT guidance.

The initial use of MRI to monitor and control thermal ablation was reported by Jolesz et al. [20]. They used MRI for preoperative targeting of the lesion and for postoperative demonstration of reversible and irreversible thermal changes of the Nd:YAG laser on tissues. However, they could not use MRI to predict the tissues’ actual temperature change during the ablation procedure [20]. Recent advances in MRI equipment, thermal imaging sequences, software, and laser delivery techniques and equipment enabled the prediction and accurate control of tissue temperatures which renewed the use of the Nd:YAG laser. This reintroduced the laser as a promising minimally invasive alternative for management of several intracranial pathologies. Details about the mechanism of action and different neurosurgical clinical uses are described below.

### Mechanism of action

The principle of LITT is selective ablation of tumor cells by heat. The laser is selectively applied to the region of the tumor using optical fibers. LITT uses an Nd:YAG laser with a wavelength of 1064 nm. The tissue penetration ranges from 2 to 10 mm [21]. When the laser hits the tumor, the tumor tissue interacts by absorbing the laser photons, which are then transformed into thermal energy insider the tumor tissue. When the temperature of the tissue is between 43 °C and 45 °C for more than 10 min, the cancer cells are sensitized to chemotherapy and radiation therapy. When the temperatures ranges between 50 °C and 80 °C for a shorter amount of time, tumor necrosis occurs through protein denaturation [22]. The thermally induced tissue damage depends on the temperature in the treated tissue and the total time the thermal energy is applied. The damage can be quantified using the Arrhenius thermal dose model, which estimates the tissue damage in relation to a thermal model where complete tissue necrosis occurs between 25 and 240 min at 43 °C [22, 23]. Using this model, MRI software can generate thermal maps to visualize thermal changes and monitor tumor necrosis [22, 24, 25].

Monitoring the thermal changes in the lesion during ablation is essential to ensure complete ablation of the lesion. CSF spaces or a blood vessel close to the lesion can dissipate the heat away from the ablated lesion due to a heat sink effect. Although this effect can lead to incomplete ablation, it can also act as an insulator protecting a nearby vital structure from the thermal injury generated at the ablation zone [26–28]. Monitoring thermal changes in fat containing lesion is challenging. The effect of the chemical composition of fat on the MRI sequences used in thermal monitoring makes it less susceptible to temperature changes leading to erroneous temperature readings [28].

### LITT system

The LITT system comprises a laser system, workstation, and MRI. There are two clinically U.S. Food and Drug Administration (FDA)–approved LITT ablation systems in the United States: Visualase (Visualase, Inc.) and NeuroBlate (Monteris Medical, Inc.). The main differences between the two systems are the laser wavelength, cooling method, heat production, and distribution pattern. The NeuroBlate system, approved by FDA in 2009, has a 1064-nm diode pulsed laser with a CO_2_-cooled side-firing probe or diffusing tip probe. The Visualase system, approved by the FDA in 2007, has a 980-nm diode continuous laser with a saline cooled diffusing applicator tip.

#### Laser

The laser system comprises a laser light source, laser fibers, applicator, sheath and diffusion tip [29]. The laser is generated by the source and then transmitted from the source to the tumor through optical fibers [29]. During transmission of the laser, part of the energy can be lost and is absorbed by the transmitting fibers, which can eventually damage the fibers [21]. Laser fibers can be optical or sapphire. Sapphire fibers are better, as they are heat resistant and transmit lasers with minimal energy absorption, making them more durable and efficient. The laser fibers are flexible and are carried to the center of the tumor by an applicator system (Visualase) or a self-contained system (Neuroblate). Different types of applicators exist; however, a cooled tip is the most useful for LITT. Cooling allows the ablation to continue for longer and at higher temperatures without damaging the diffusing tip or charring the tumor tissue on contact [29, 30]. Charring decreases the absorption of laser energy and interferes with transmission of heat [31]. An optical diffusing tip modifies the laser beam to a spherical emission, hence achieving a homogenous and symmetric distribution of energy into a sphere of tissue [32].

#### Workstation

MRI images obtained before and during the LITT procedure are sent from the MRI scanner to a linked workstation. The workstation provides real-time thermal maps for monitoring the procedure and estimates tissue necrosis. With the Visualase system, one can also assign temperature limits as safety points to trigger system deactivation, preventing undesired thermal damage to nearby vital organs or surrounding structures [24, 25, 33]. The Neuroblate system has a thermocouple at the tip of the probe that determines the baseline brain temperature and then regulates the amount of CO_2_ circulated through the tip of the probe to maintain a predetermined temperature range. The laser shuts off automatically if the valid temperature range at the tip is exceeded [34].

#### MRI

Successful thermal ablation requires accurate targeting of the tumor and maintenance of a sufficient temperature level while excluding damage to the adjacent structures [22, 35]. MRI is used to identify the lesion and plan the trajectory for the laser probe [3]. More importantly, it is used to visualize and quantify heat deposition within and surrounding the area of ablation, a process called magnetic resonance thermometry. MR thermometry provides a noninvasive, real-time temperature monitoring during the procedure and assesses target cell death [22]. MRI thus is essential to the safety and efficacy of the procedure [22].

### Technique

The first step of LITT is preprocedural stereotactic MRI [3]. A post-gadolinium axial spoiled gradient volumetric sequence is acquired and used for registration and to ensure adequate delineation of the tumor. This imaging can also be obtained via intraoperative MRI immediately before the LITT procedure. The patient is put in a lateral, supine, or prone position depending on the tumor location, and general anesthesia is administered in the operating room. Navigation software is used for registration and trajectory planning. The operator determines the appropriate entry point, the target, and the trajectory angle. The ideal trajectory should avoid, when possible, passing through scar tissue, the operative bed, the ventricles, vessels at the entry point, and angulation at the entry point should not exceed 30°.

Our institution currently uses the Neuroblate ablation system for intracranial procedures. A burr hole is made at the entry site, and a stereotactic bolt is placed in the calvarium for one trajectory, or two bolts are placed for two trajectories for larger masses. The patient is then positioned within our intraoperative MRI. A robotic probe driver is attached to the stereotactic bolt or bolts. The laser probe or probes are then advanced through the hole until they reach the center of the lesion. Before starting the ablation, pretreatment images are obtained. 3D T1-weighted fast spoiled gradient images with a small field of view are acquired to show the full length of the probe and to ensure accurate positioning in the lesion. A T1 or T2-FLAIR image is then acquired to act as an anatomical reference image as a background on the workstation to overlay the real-time thermal images. Thermal imaging uses a fast spoiled gradient recalled echo sequence, which takes about 8 s, and is run repeatedly during the ablation procedure. When ablation starts, the acquired images are compared with the reference images at the workstation and generate color-coded thermal maps. Using the Arrhenius model, based on ablation time and temperature, the workstation generates an irreversible damage estimate map, which is a color-coded image that overlays the reference image. The laser is stopped when the estimated irreversible damage extends to include the entire desired ablation area. A post-LITT subtraction scan and dynamic contrast-enhanced (DCE) perfusion scan can be obtained to evaluate the extent of ablation and whether there is any residual untreated tumor [34].

### MRI features and histologic changes

The MRI appearance of the ablated lesion depends on the time of imaging in relation to the ablation procedure. During the ablation, the effect of the laser-induced thermal energy on the target tissue may have a characteristic zonal architecture [36, 37], which can be seen up to 3 months following the procedure. Later, the zonal organization becomes less conspicuous [36, 38–40].

#### Immediate and early stage (0 to 3 months post procedure)

**Fig. 1:**
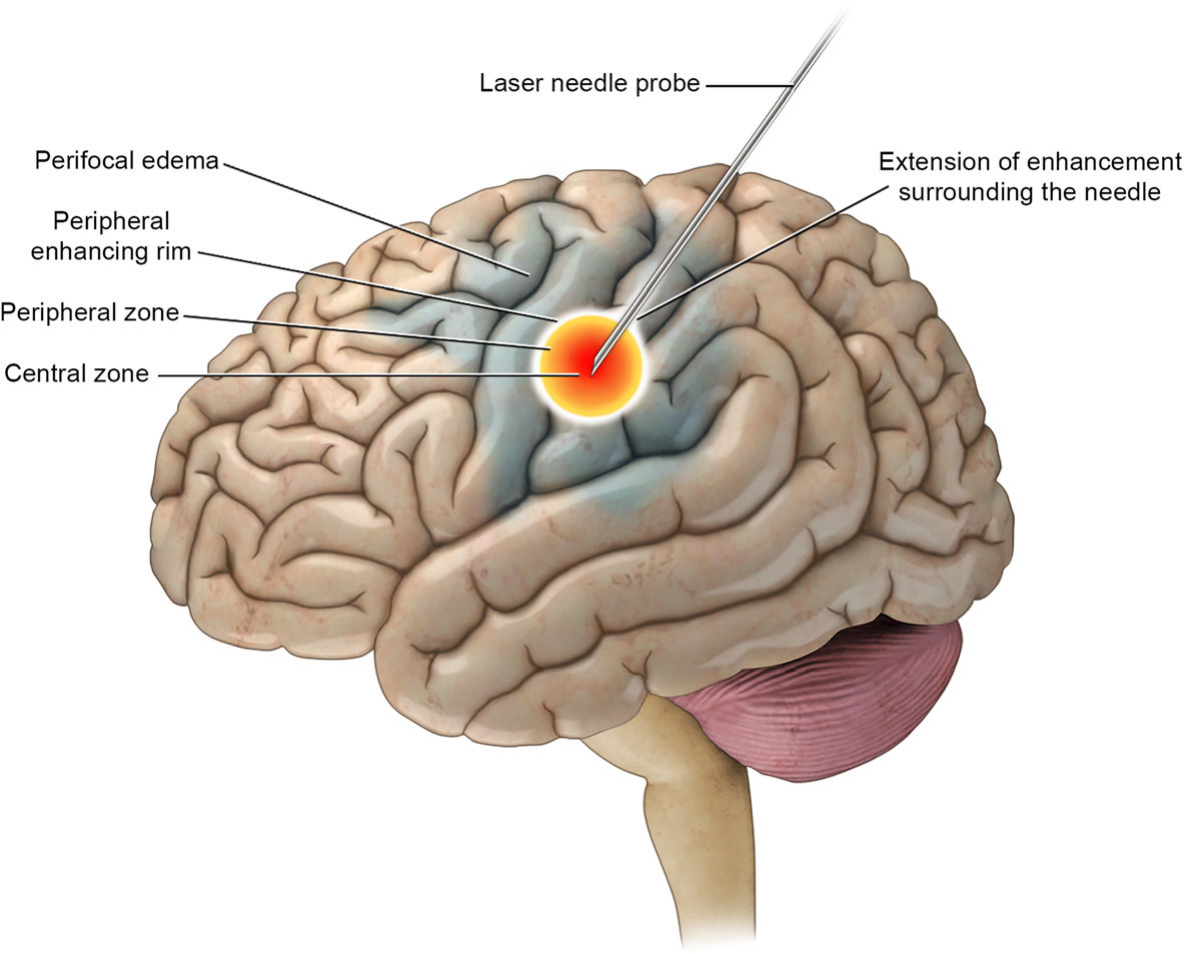
Immediately after and in the early stages after LITT (0 to 3 months post procedure). The treated lesion shows a distinct central zone and peripheral zone surrounded by vasogenic edema

Two zones surround the laser tip appear around the lesion: a central and a peripheral zone. The central zone, where early liquefactive necrosis occurs, immediately surrounds the laser probe. The peripheral zone, where edema with irreversible cell damage occurs, lies immediately outside the central zone. Outside the peripheral zone are reversible parenchymal perilesional edema and viable cells [36, 40]; and contrast leakage, due to the breakdown of the blood-brain barrier, is often noted (Fig. 1, Table 1).

1. Central zone:
  - MRI features: When the ablation process begins, the central zone exhibits hyperintense T1-weighted and hypointense T2-weighted image signals. The T1 hyperintensity increases over time and either plateaus or continues to increase until the end of the procedure [39].
  - Size: The diameter of the central zone also increases over time and either plateaus or continues to increase until the end of the procedure [39].
  - Histology: The histologic appearance of the central zone corresponds well with the MRI signal. Initially, there is damage of the cellular and subcellular membranes including the nuclear membrane and mitochondria of the nerve cells, glial cells and endothelium [38]. These membranes are either damaged or become fragmented. The blood vessels are engorged and contain grouped red blood cells that have cell membrane defects. Hemoglobin escape through these defect and the erythrocyte becomes empty [36, 38]. The increasing T1 hyperintensity of the central zone could be due to hemoglobin degradation product leaking from the defective red blood cells’ membranes [39]. Immediately after LITT, the structural tissue damage is minimal. In the early stages after the procedure, the necrosis becomes apparent, and resorptive changes start from the periphery of the central zone [36].
2. The peripheral zone:
  - MRI features: The peripheral zone is simply a zone of edema exhibiting hypointense T1-weighted and hyperintense T2-weighted imaging signals. The T1 hypointensity increases with time. A thin enhancing rim, seen in the post-ablation MRI, at the margin of the peripheral zone defines the total volume of thermally induced cell damage [39]. This rim can extend to the laser catheter track on future follow-up studies [39]. The enhancement may be due to a disrupted blood-brain barrier from the damaged blood vessels [39].
  - Size: The diameter of the peripheral zone increases over time. The total size of the ablated lesion includes the central and peripheral zones [39].
  - Histology: There is intracellular edema. Granulocytes, lymphocytes, and macrophages infiltrate this layer. A layer of reactive astrocytes surrounds this area and separate it from the normal brain tissue [38]. The peripheral zone is called the zone of necrotizing edema, as these changes are irreversible and the cells are not viable [36, 39]. The outer thin enhancing rim comprises damaged blood vessels [41] and granulation tissue from the adjacent viable tissue [36].

**Table 1:** MRI of laser-ablated lesion: Immediate and early stage (0 to 3 months post procedures)

Structural anatomy	Histology	MRI features		
		T1WI	T1WI + C	T2WI
Central zone	Coagulative necrosis (damage of nuclear membrane and mitochondria, engorged blood vessels, RBCs with cell membrane defects and no Hemoglobin)	Hyper	None	Hypo
Peripheral zone	Necrotizing edema (intracellular edema, ↑granulocyte, lymphocyte, and macrophages)	Hypo	None	Hyper
Outer rim bordering the peripheral zone	Damaged blood vessels and granulation tissue	Hypo/Hyper	Enhanced	Hypo
Perifocal edema (outside the peripheral zone)	Vasogenic edema with viable cells	Hypo	None	Hyper

*RBC* red blood cell, *T1WI* T1-weighted image, *T2WI* T2-weighted image, *C* contrast, *hyper* hyperintense, *hypo* hypointense

#### Delayed stage and follow-up (2 weeks to 6 months post procedure)

Within the first 2 weeks after ablation, the lesion initially grows. However, it shrinks later on. The T1 hyperintense signal of the central zone decreases, and the T1 hypointense signal of the peripheral zone increases, making the lesion more homogenous and the zonal organization less conspicuous [39]. The enhancing rim at the border of the peripheral zone persists but decreases in size and enhancement [39, 40], and finally, a spot-like residual enhancement can be seen up to 4 years after the procedure [40] (Table 2).

**Table 2:** MRI of laser-ablated lesion: Delayed stage (2 weeks to 6 months post procedure)

Structural anatomy	MRI features	
	T1WI	T1WI + C
Central zone	↓ Hyper	None
Peripheral zone	↑ Hypo	None
Outer rim bordering the peripheral zone	Hypo/Hyper	↓ Enhancement (size and degree)
Perifocal edema (outside the peripheral zone)	Hypo	Hyper

*T1WI* T1-weighted image, *C* contrast, *hyper* hyperintense, *hypo* hypointense

The perilesional edema is located beyond the peripheral zone. It can be separated from the ablated lesion on imaging by the enhancing rim bordering the peripheral zone in the post-contrast T1-weighted image with corresponding T2-weighted image hypointense rim. The perilesional edema may not develop immediately after the procedure; it usually starts 1 to 3 days after ablation and can show mild to severe progression, easily assessed on T2-weighted imaging. The perilesional edema is reversible and usually resolves over the course of 2 to 9 weeks [39, 40].

### Applications

Several studies over the past 2 decades have addressed the use of LITT to treat a variety of cerebral pathologies and have established the feasibility and safety of the technique. In addition, these studies identified potential indications for LITT and revealed complications that can occur. However, these studies could not assess the added survival benefit of LITT compared with that of other available methods of treatment. There was selection bias, as the procedure was performed in selected groups of patients, and studies were not randomized or controlled. There were many confounding factors, as several studies had different pathologies and many patients may have had multiple pathologies and received various treatments either before or after the procedure, ultimately affecting their survival. Also, a small number of patients were studied, and several of the studies were case reports or case series. Despite the lack of information on survival and the aforementioned limitations, the current literature demonstrates a variety of common applications for LITT that have been observed to lead to successful elimination of lesions and treatment of other conditions. The various clinical trials published to date as well as their outcomes are summarized in Table 3.

**Table 3:** Summary of studies reporting clinical application of LITT in neurosurgery

Reviewed studies	Number of Cases	Indications for LITT	Outcome	Comments
Schwarzmaier et al. [32]	16; 2 sets of patient (10 + 6)	Recurrent glioblastoma	Median survival time: 5.2 for the first set, and 11.2 in the second set	Learning curve deemed responsible explaining different survival
Carpentier et al. [33]	4	Recurrent glioblastoma	Mean overall survival: 10.5 months	Three complications: transient dysphasia, seizure, and cerebrospinal fluid leak
Jethwa et al. [3]	20	Multiple primary brain tumors	No data about survival was provided	Four complications: arterial injury, refractory brain edema, pituitary injury, and misplacement of the laser probe
Banerjee et al. [2]		Recurrent grade III/IV glioblastoma	Median overall survival after LITT: 20.9 months, improved compared to other treatment modalities	
Rao et al. [46]	14	Recurrent brain metastases after radiosurgery and/or whole-brain radiation	Median progression-free survival: 37 weeks, and overall survival: 57%	
Carpentier et al. [44, 45]	2 studies: 2008: 4 2011:7	Recurrent or resistant cerebral metastases	2008: Not reported 2011: follow-up up to 30 month, median survival was 19.8 months	
Bastos et al. [60]	61	Recurrent brain metastasis and radiation necrosis	Incomplete ablation and recurrent tumoral lesions were associated with a higher risk of treatment failure and were the major predicting factors for local recurrence Systemic therapy within 3 months after LITT was a protective factor against local recurrence	
Kang et al. [64]	20	Epilepsy	LITT achieved a 53% rate of remission of disabling seizures	
Waseem et al. [65]	7	Epilepsy	LITT achieved a 57% rate of remission of disabling seizures	
Willie et al. [26]	13	Epilepsy	LITT achieved a 54% rate of remission of disabling seizures	

### Common LITT applications

#### Primary brain neoplasm

The majority of the studies regarding LITT involve treatment of primary brain neoplasms, including gliomas. Schwarzmaier et al. [32] investigated survival after LITT in 16 patients with recurrent glioblastoma, divided into 2 sets of patients over 2 time periods. The median survival time after recurrence was 5.2 months in 2001–2002 (10 patients) and 11.2 months in 2003–2004 (6 patients). The authors attributed the increased survival in 2003–2004 to 2 factors: a learning curve from the first set of patients may have influenced the second set, and the interval between the diagnosis of recurrence and the LITT was longer for the first set (2 months) than for the second (0.3 months). The only complication due to the LITT procedure was transient arm weakness in 1 patient.

In 2012, Carpentier et al. [33] did a pilot study in 4 patients with recurrent glioblastoma to investigate survival benefits after LITT. In all 4 patients, recurrence occurred after LITT, at a mean of 37 days, and the mean overall survival was 10.5 months. The authors reported 3 complications: transient dysphasia that resolved by the 7th day after LITT, generalized seizure on the 9th day after LITT, and cerebrospinal fluid leak on the 7th day after LITT. Recurrences were at the LITT site in 2 patients, even though the ablation extended beyond the initial tumor volume seen on the pretreatment post-contrast MRI.

The feasibility and technical aspects of MRI-guided LITT for treatment of a variety of brain pathologies were addressed by Jethwa et al. [3]. The study included 20 patients with a variety of pathologies, including 6 patients with glioblastoma, 1 patient with anaplastic astrocytoma, 3 patients with ependymoma, 2 patients with meningioma, 2 patients with hemangioblastoma, 1 patient with primitive neuroectodermal tumor, 1 patient with chordoma, and 3 patients with brain metastases. The authors reported 4 complications: arterial injury, refractory brain edema, pituitary injury, and misplacement of the laser probe. The study did not report the final outcome or survival benefits.

**Fig. 2:**
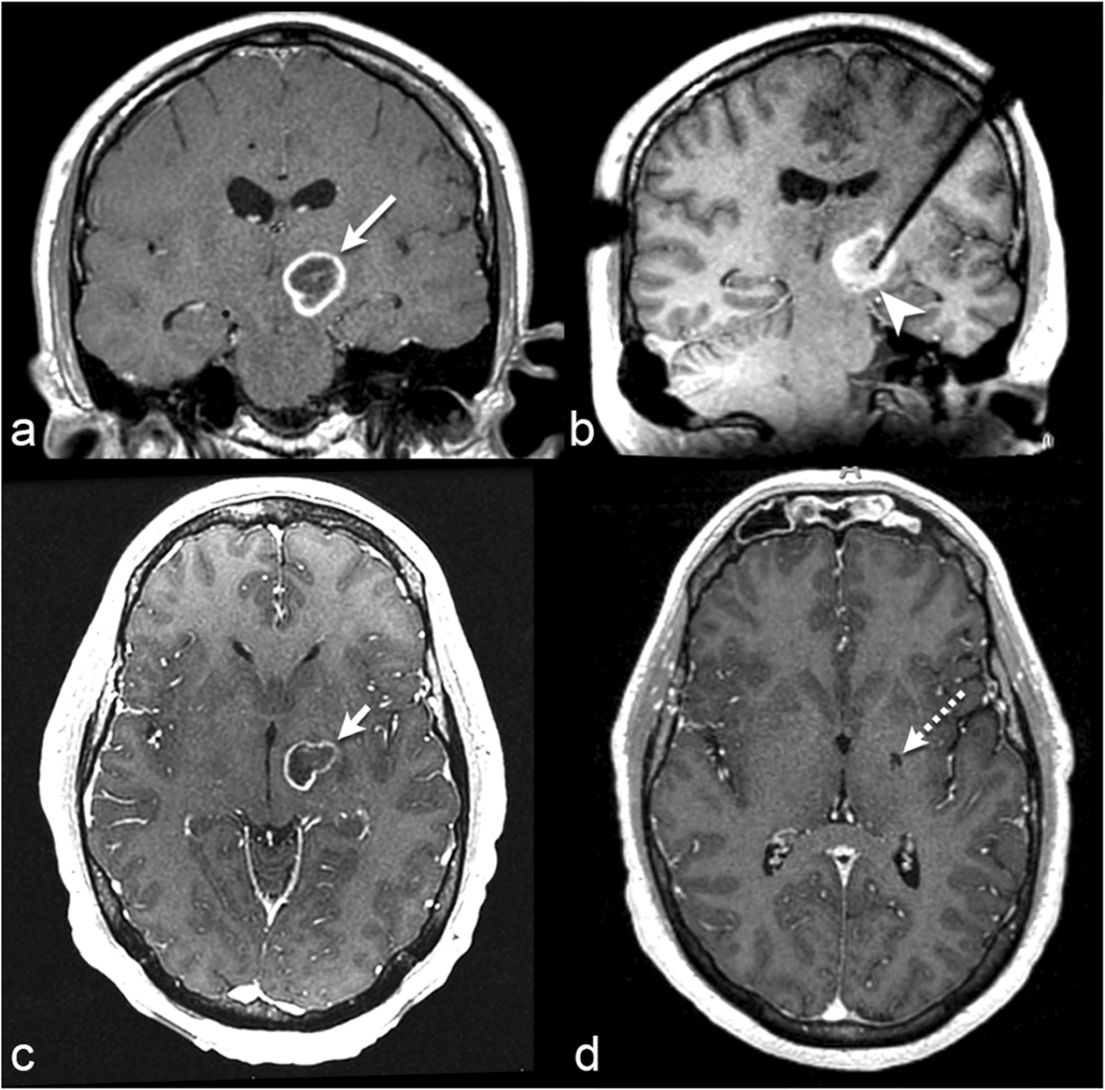
35-year-old with biopsy-proven left thalamic glioblastoma. **a** Coronal post-contrast T1-weighted MRI before LITT demonstrates a ring-enhancing mass (long arrow). **b** Coronal intraoperative localizing T1-weighted MRI shows the laser probe within the mass (arrowhead). **c** Axial post-contrast T1-weighted MRI 2.5 months after LITT shows a mild decrease in size of the mass (short arrow). **d** Axial post-contrast T1-weighted MRI 4 months after LITT demonstrates complete resolution of the glioblastoma (dashed arrow)

In 2015, Banerjee et al. [2] reviewed the literature for MRI-guided LITT in neuro-oncology to compare the outcomes and safety of LITT with those of the standard treatment used to treat each pathology. In patients with recurrent grade III/IV glioblastoma, they found that the median overall survival from the diagnosis of recurrence was improved, at 20.9 months, in patients treated with LITT compared with patients given other treatment options [42, 43]: 11.1 to 16 months for chemotherapy, 14.8 months for open surgery, 18.9 months for high-dose brachytherapy, and 24.4 months for repeated open surgery. The rate of significant procedure complications was 16.7% and included hemorrhage, permanent neurologic deficits, and infection. However, future prospective studies are needed to accurately evaluate the LITT outcome in patients with primary brain neoplasms. We provide an example of a patient with brain metastases who was successfully treated at our intuition using LITT (Fig. 2).

#### Metastases

**Fig. 3:**
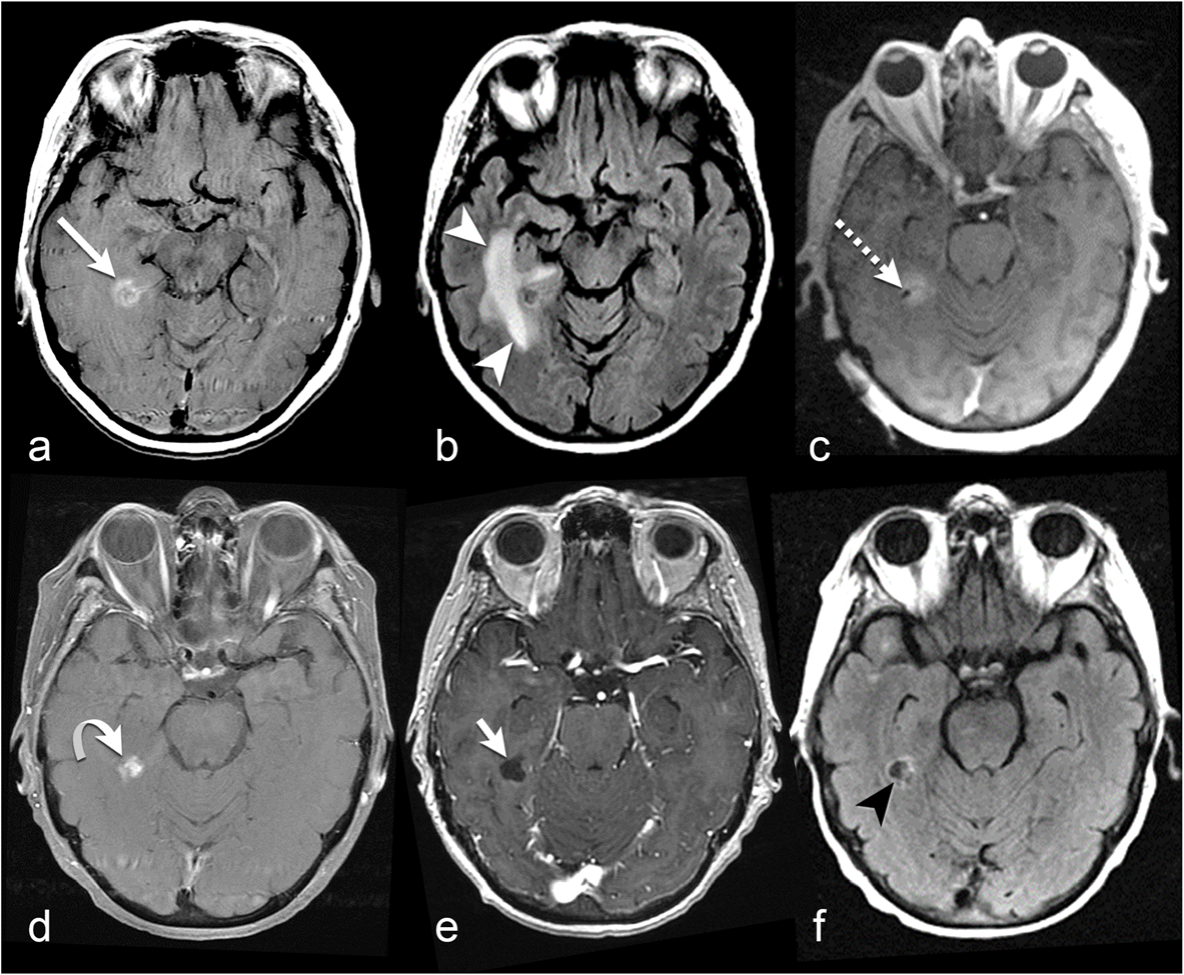
67-year-old with metastatic renal cell carcinoma. **a** Axial post-contrast T1-weighted imaging before LITT demonstrates a ring-enhancing metastasis in the right medial temporal lobe (long arrow). **b** FLAIR before LITT demonstrates surrounding vasogenic edema (white arrowheads). **c** Axial post-contrast T1-weighted imaging shows the ablation probe tip within the metastatic lesion (dashed arrow). **d** Axial post-contrast T1-weighted imaging 4 months after LITT demonstrates slightly decreased enhancement at the treated lesion (curved arrow). Axial post-contrast T1-weighted imaging (**e**) and FLAIR (**f**) 10 months after LITT show complete resolution of enhancement (short arrow) and vasogenic edema (black arrowhead)

LITT has been used in patients with metastases resistant to or recurring after standard therapy, including chemotherapy, stereotactic radiosurgery, and whole-brain radiation [44–46]. Studies of this use of LITT include case reports, case series of brain metastases, and mixed studies of both metastases and other cerebral lesions [3, 47]. Rao et al. [46] evaluated 14 patients with recurrent brain metastases after radiosurgery and/or whole-brain radiation; the metastases were from lung cancer primaries in 11 patients, breast cancer in 2 patients, and colon cancer in 1 patient. In 2 pilot clinical trial studies, Carpentier et al. [44, 45] investigated the feasibility and effectiveness of LITT in patients with recurrent or resistant cerebral metastases: a study in 2008, of 4 patients with metastases from lung and breast primaries and a study in 2011 of 7 patients. In both studies, the author reported the success of the procedure without complications. After a literature review, Banerjee et al. [2] found that among patients with metastases from lung or breast primaries, the median overall survival was 12.6 months and ranged from 9.0 to 19.8 months after LITT, in contrast to a median survival of 7.0 to 28.6 months in patients treated with stereotactic radiosurgery with or without whole-brain radiation [48–50]. However, progression-free survival [2] ranged from 3.8 to 8.5 months after LITT but was 26.4 months after other treatments. The rate of severe complications was 8% with LITT [2] and 10 to 13% with open surgery. Although the overall survival and progression free survival shows no significant added survival benefits for the patients treated with LITT compared to the standard therapy; the author had study limitations which may have affected his conclusions. The studies he reviewed were not homogenous and had different inclusion and exclusion criteria. In addition, several studies did not report the recurrence rate after LITT. We provide an example of a patient with brain metastases who was successfully treated at our intuition using LITT (Fig. 3).

#### Radiation necrosis

Radiation necrosis is a complication of radiation therapy where irreversible, sometimes progressive necrosis occurs months to years after completion of radiation therapy [51, 52]. The incidence of radiation necrosis varies from 3 to 24% [53, 54]. However, the literature might not reflect the actual incidence of radiation necrosis, as it is difficult to differentiate from tumor recurrence. In addition, histopathologic assessment of the suspected radiation necrosis was not performed for all patients studied. The risk of radiation necrosis increases significantly with an increased radiation dose or fraction size or with subsequent chemotherapy [54]. Several mechanisms for this radiation injury have been proposed, including vascular injury leading to increased capillary permeability and edema, glial and white matter damage, and immune mechanisms [51]. Recently, it was suggested that the astrocytes surrounding the irradiated necrotic tissue become abnormal and produce high-concentration vascular endothelial growth factor (VEGF), which is linked to the pathogenesis of radiation necrosis through angiogenesis and increased capillary permeability, leading to perilesional edema [55]. The standard treatment for radiation necrosis is steroids, while surgery is reserved for refractory cases [56]. In patients with necrosis who cannot tolerate surgery or whose lesion is not accessible by surgery, LITT can play a role. The potential efficacy of LITT against radiation necrosis may be due to ablation of the abnormal, VEGF-producing astrocytes surrounding the necrosis [57]. The literature contains only few cases where LITT was used to treat medically refractory radiation necrosis [57, 58]. A limitation of these reports is a diagnosis based on imaging, including MRI, PET, CT, and MR spectroscopy, rather than tissue assessment.

One case report was of a patient with metastatic lung cancer who received stereotactic radiosurgery for brain metastases [57]. The patient presented 10 months after the radiation therapy with clinical and imaging findings of radiation necrosis. The patient did not show a response to medical treatment, and he was not a surgical candidate owing to multiple comorbidities. LITT was performed successfully, and the patient was discharged 48 h later without significant complications. The patient was weaned off steroids, and imaging 7 weeks after the procedure showed almost complete resolution of the edema [57].

In a case series of 6 patients [58], imaging was suggestive of radiation necrosis in 3 patients, tumor progression in 2 patients, and a mixture of both in 1 patient. The LITT procedure was performed successfully in all the patients. Imaging 2 weeks after the procedure showed decreased edema, and the patients were weaned off steroids by 2 months after the procedure. Four of the 6 patients achieved resolution of symptoms and decreased lesion size until death occurred after 6 months of follow-up. One patient died 1 month after LITT from systemic disease from cancer. One patient had regrowth of the lesion 3 months after treatment and underwent standard craniotomy [58].

Another report included a single patient with persistent edema following stereotactic radiosurgery of a metastatic lung cancer [59]. However, the authors said that it was not clear whether the persistent edema was due to radiation necrosis or to recurrence. The patient maintained steroid therapy until he underwent LITT 14 weeks after the stereotactic radiosurgery. The patient was weaned off steroids over the course of 2 weeks following LITT, and imaging 10 weeks after LITT showed only a small amount of vasogenic edema.

At our institution, we currently use LITT for treatment of selected patients with recurrent brain metastases and radiation necrosis following stereotactic radiosurgery. A retrospective analysis at our institution investigated survival after LITT in 61 patients with recurrent brain metastasis and radiation necrosis [60]. The study analyzed the effects of multiple factors on progression-free survival, including the extent of ablation (incomplete vs complete), dural-based status, lesion volume (> 6 cm^3^ vs < 6 cm^3^), systemic treatment before and after LITT, and nature of the lesion (radiation necrosis vs tumor recurrence). Incomplete ablation (hazard ratio 4.88, 95% CI 2.22–10.75, *p* < 0.001) and recurrent tumoral lesions (hazard ratio 2.206, 95% CI 1.024–4.753, *p* = 0.028) were associated with a higher risk of treatment failure and were the major predicting factors for local recurrence. On the other hand, systemic therapy within 3 months after LITT was a protective factor against local recurrence (hazard ratio 2.56, 95% CI 1.15–5.67, *p* = 0.021).

**Fig. 4:**
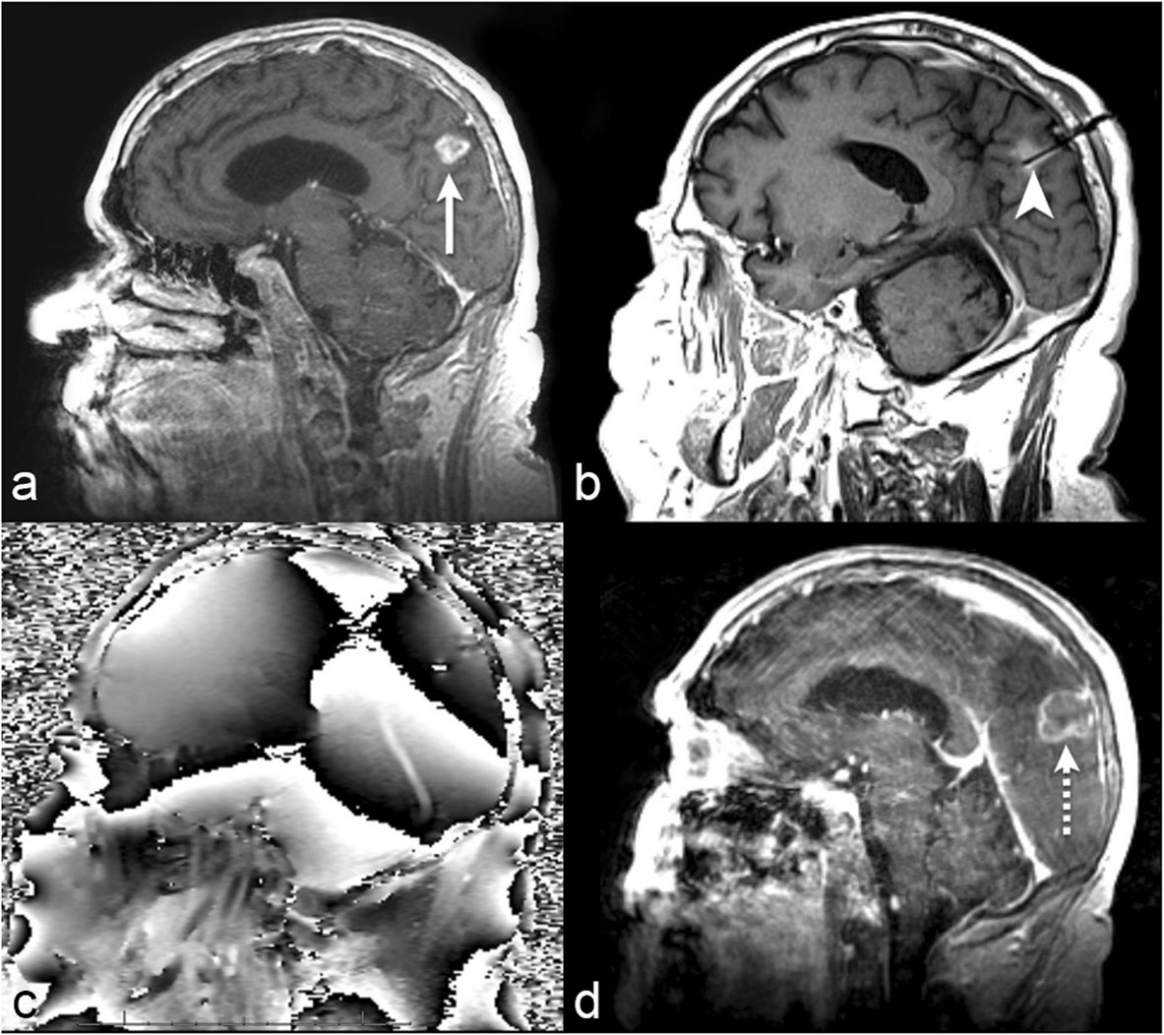
72-year-old with metastatic melanoma. **a** Sagittal post-contrast T1-weighted imaging before LITT demonstrates a progressing enhancing lesion in the left inferior parietal lobule at a site of a brain metastasis previously treated with gamma knife radiation therapy (long arrow). The lesion was biopsied intraoperatively immediately before LITT and was found to represent radiation necrosis. **b** Sagittal intraoperative localizing T1-weighted imaging with the laser probe within the lesion (arrowhead). **c** Sagittal intraoperative gradient-echo phase imaging is the source of the thermography maps. By subtracting subsequent images during heating from a reference image acquired before heating, a map of temperature change can be formed. **d** Sagittal post-contrast T1-weighted imaging 1 month after LITT demonstrates complete ablation of the lesion (dashed arrow)

Another retrospective analysis at our institution investigated response after LITT in 36 patients with brain metastasis that progressed despite treatment with stereotactic radiosurgery [61]. The study showed a significant difference in the effects of pre-treatment lesion volume on response to LITT (*p* = 0.012). The mean pre-treatment volume of the brain metastases was 5.05 cc (Range- 0.54 to 23.31). Smaller tumor volumes (mean volume was 3.54 cc and range was 0.54 cc to 10.06 cc) responded to LITT compared to larger tumor volumes (mean volume was 8.81 cc and range was 0.93 cc to 23.31 cc). The author reported 16 out of 36 patients experienced post-operative neurological complications [61]. We provide an example of a patient with radiation necrosis who was successfully treated at our intuition using LITT (Fig. 4).

#### Epilepsy

Patient with medication-resistant epilepsy can experience disabling seizures affecting their quality of life. For these patients, surgical resection of the epileptogenic focus can eliminate these seizures [62]. For example, anterior temporal lobectomy or selective amygdalohippocampectomy can eliminate seizures in 75% of patients with mesial temporal lobe epilepsy [62]. However, the surgical approach for a deeply seated epileptogenic focus can damage adjacent important structures, causing significant morbidity [62]. Laser ablation is a promising alternative to surgery, as it can selectively ablate the epileptic focus while avoiding collateral damage caused by surgery [63, 64]. Several studies have investigated the feasibility, effectiveness, and possible complications of this technique. The procedure was shown to be safe and feasible; however, the likelihood of eliminating seizures was lower than for surgery [65]. In a series of 20 patients with medication-resistant epilepsy, LITT achieved a 53% rate of remission of disabling seizures [65]. This result was similar to another study that included 7 patients with temporal lobe epilepsy, where 57% of patients were either seizure free or disabling seizure free [66]. Another study with 13 patients showed 54% remission at 6 months, which improved to 61% after 12 months [26].

#### Chronic pain

Patients with cancer may experience chronic pain, which may be severe or persistent. These patients are referred to neurosurgeons after all medical treatment options are exhausted. The neurosurgeon targets the pain centers or pain pathways to interfere with pain transmission. Radiofrequency thermocoagulation of the cingulum, the pain center in the brain, is a minimally invasive procedure, performed in critically ill or nonsurgical patients using only local anesthesia [26]. However, the procedure provides only temporary relief and may not be effective in all patients [67]. MRI-guided LITT of the cingulum has been tried as an alternative to radiofrequency ablation with the advantage of MRI guidance and real-time thermal monitoring to ensure the accuracy of ablation and to avoid collateral damage [68]. However, the effectiveness and benefits of MRI-guided LITT in management of chronic pain are unknown owing to the small number of patients and lack of reported outcomes in the literature.

### Complications and unfavorable outcomes

LITT is a less invasive procedure with a very low incidence of complications compared to open surgery. Jethwa et al. [3] and Pruitt et al. [27] reported few potential complications in a series of 20 and 46 patients respectively with brain neoplasms treated with LITT and produced recommendations to help avoid or lessen these complications. The complications and the recommendations to avoid are summarized in Table 4.

**Table 4:** Summary of common complications of LITT and recommendation to avoid

	Reported complication	Recommendation	Reference
1	Arteria injury leading to hemorrhage	• Choosing the safer trajectory even if it is longer (Jethwa et al.)	Jethwa et al. (1 patient), Pruitt et al. (3 patients)
2	Refractory brain edema due to large lesion size	• Ideal lesion: < 3 cm in diameter, well defined. • Staged procedure for larger lesion. • Pre procedure steroids (Jethwa et al.)	Jethwa et al. (1 patient)
3	Thermal injury to a nearby vital structure	• Using smaller diffusing tips. • Use caution when target lesion is not adjacent to CSF space (act as a protective heat sink) (Pruitt et al.)	Jethwa et al. (1 patient), Pruitt et al. (3 patients)
4	Catheter malposition	• Using an alignment rod. • Avoid use of plastic skull anchors (Pruitt et al.)	Jethwa et al. (1 patient), Pruitt et al. (4 patients)

Other complications of LITT reported in the literature include permanent neurologic deficit, parenchymal infection, transient focal neurologic deficit, seizures, and cerebrospinal fluid leak. In a study of 102 patients with different pathologies treated with LITT, death was reported in 3 patients. The cause of death was refractory post-procedure edema in one patient and rapid disease progression in the other 2 patients. Whether the rapid disease progression was related to the procedure was not discussed [68].

### Novel research in progress

Recent research is developing the use of gold nanoshells to aid thermal ablation in achieving selective destruction of tumor cells. Nanoshells have a spherical core and thin metal coat [69]. The nanoshell can absorb the light in the near infrared and transform it into heat, leading to local cell destruction. Once these particles localize to the tumor cell, they increase the heat sensitivity of the tumor cells to the laser compared with adjacent nanoshell-lacking tissues, which increases the thermal effects on the tumor without increasing the harmful thermal effects to the surrounding tissues [70]. The first step is to generate a nanoshell that can absorb the laser used during the procedure with the highest efficiency. The ability of the nanoshell to absorb light at a specific wavelength is determined by the nanoshell size and composition. With a silica core of 100 nm coated with a 10-nm gold shell, the nanoshell will have peak light absorption near the infrared range [71]. The second step is to localize the nanoparticle in the tumor cells. This localization can be achieved by choosing a nanoshell size that can pass only through the “leaky” tumor vessels [72]. Another method to target the nanoshell into the tumor is by specific vectors, such as macrophages and tumor receptor–specific antibodies [73, 74]. Recently, studies have shown that chemotherapy can be incorporated into the nanoshell, and laser-generated heat can rupture the nanoshell and selectively liberate the chemotherapy within the cancer cells [75].

## Conclusions

LITT is a less invasive treatment modality with a lower incidence of complications compared to open surgery. The principle of LITT is selective ablation of tumor cells by heat and is monitored by real-time MRI thermometry. LITT has a range of applications, such as treatment of glioma, metastases, radiation necrosis, chronic pain, and epilepsy. LITT is used for selected lesions and in selected patients as a safer alternative treatment option for patients in whom the lesion is not accessible by surgery, in patients who are not surgical candidates, or in those in whom other standard treatment options have failed. Complications of LITT include hemorrhage, brain edema, thermal injury of adjacent structures, and treatment failure. Reported rare complications include permanent neurologic deficit, brain parenchymal infection, transient focal neurologic deficit, seizures, and cerebrospinal fluid leak. A learning curve and increased operator experience was observed by some authors to decrease the incidence of complications.

Although the current literature did not provide significant and/or accurate survival benefits, it establishes the feasibility of the procedure and helps explore the potential indications and possible complications. Future prospective randomized controlled clinical trials with a larger number of patients and adequate follow-up periods are needed to determine patient outcomes and evaluate survival benefits.

## Data Availability

Not applicable.

## Abbreviations

LITT: Laser interstitial thermal therapy
MRI: Magnetic resonance imaging
Nd:YAG: Neodymium-doped yttrium aluminum garnet
VEGF: Vascular endothelial growth factor

## References

[1] Norred SE, Johnson JA (2014). Magnetic resonance-guided laser induced thermal therapy for glioblastoma multiforme: a review. Biomed Res Int.

[2] Banerjee C, Snelling B, Berger MH, Shah A, Ivan ME, Komotar RJ (2015). The role of magnetic resonance-guided laser ablation in neurooncology. Br J Neurosurg.

[3] Jethwa PR, Barrese JC, Gowda A, Shetty A, Danish SF (2012). Magnetic resonance thermometry-guided laser-induced thermal therapy for intracranial neoplasms: initial experience. Neurosurgery.

[4] Fine S, Klein E, Nowak W, Scott RE, Laor Y, Simpson L, Crissey J, Donoghue J, Derr VE (1965). Interaction of laser radiation with biologic systems. I. Studies on interaction with tissues. Fed Proc.

[5] Fox J, Hayes J, Stein M, Green R (1967). Effects of laser radiation on intracranial structures.

[6] Fox JL, Hayes JR, Stein MN, Green RC, Paananen R (1967). Experimental cranial and vascular studies of the effects of pulsed and continuous wave laser radiation. J Neurosurg.

[7] Krishnamurthy S, Powers SK (1994). Lasers in neurosurgery. Lasers Surg Med.

[8] Stellar S (1967). A study of the effects of laser light on nervous tissue.

[9] ROSOMOFF HL, CARROLL F (1966). Reaction of neoplasm and brain to laser. Arch Neurol.

[10] Stellar Stanley, Polanyi Thomas G., Bredemeier Herbert C. (1974). Lasers in Surgery. Laser Applications in Medicine and Biology.

[11] Andrews AH, Polanyi TG, PW A (1982). Neurosurgery. Microscopic and endoscopic surgery with the CO2 laser. edn.

[12] Kaplan I, PW A (1978). The use of CO2 in neurosurgery. Laser surgery II. edn.

[13] Heppner F, Kaplan I (1978). The laser scalpel on the nervous system. Laser surgery II. edn.

[14] Stellar SPT, Bredemeier HC, Wolbarsht ML (1971). Lasers in surgery. Laser applications in medicine and biology. Volume 1, edn.

[15] Ognev B, Vishnevskii A, Troitskii R, Timokhina N (1972). Changes in the brain and eyes produced by lasers. Bull Exp Biol Med.

[16] Beck O, Wilske J, Schönberger J, Gorisch W (1979). Tissue changes following application of lasers to the rabbit brain. Neurosurg Rev.

[17] Bown S (1988). The future of lasers in cancer therapy. Br J Hosp Med.

[18] Bown S (1983). Phototherapy of tumors. World J Surg.

[19] Sugiyama K, Sakai T, Fujishima I, Ryu H, Uemura K, Yokoyama T (1990). Stereotactic interstitial laser-hyperthermia using Nd-YAG laser. Stereotact Funct Neurosurg.

[20] Jolesz FA, Bleier AR, Jakab P, Ruenzel PW, Huttl K, Jako GJ (1988). MR imaging of laser-tissue interactions. Radiology.

[21] Mensel B, Weigel C, Hosten N (2006). Laser-induced thermotherapy. Recent Results Cancer Res.

[22] Rieke V, Butts Pauly K (2008). MR thermometry. J Magn Reson Imaging.

[23] Chang IA (2010). Considerations for thermal injury analysis for RF ablation devices. Open Biomed Eng J.

[24] McNichols RJ, Gowda A, Kangasniemi M, Bankson JA, Price RE, Hazle JD (2004). MR thermometry-based feedback control of laser interstitial thermal therapy at 980 nm. Lasers Surg Med.

[25] McNichols RJ, Kangasniemi M, Gowda A, Bankson JA, Price RE, Hazle JD (2004). Technical developments for cerebral thermal treatment: water-cooled diffusing laser fibre tips and temperature-sensitive MRI using intersecting image planes. Int J Hyperth.

[26] Willie JT, Laxpati NG, Drane DL, Gowda A, Appin C, Hao C, Brat DJ, Helmers SL, Saindane A, Nour SG (2014). Real-time magnetic resonance-guided stereotactic laser amygdalohippocampotomy for mesial temporal lobe epilepsy. Neurosurgery.

[27] Pruitt R, Gamble A, Black K, Schulder M, Mehta AD (2017). Complication avoidance in laser interstitial thermal therapy: lessons learned. J Neurosurg.

[28] Medvid R, Ruiz A, Komotar RJ, Jagid JR, Ivan ME, Quencer RM, Desai MB (2015). Current applications of MRI-guided laser interstitial thermal therapy in the treatment of brain neoplasms and epilepsy: a radiologic and neurosurgical overview. AJNR Am J Neuroradiol.

[29] Stafford RJ, Fuentes D, Elliott AA, Weinberg JS, Ahrar K (2010). Laser-induced thermal therapy for tumor ablation. Crit Rev Biomed Eng.

[30] Vogl TJ, Mack MG, Roggan A, Straub R, Eichler KC, Muller PK, Knappe V, Felix R (1998). Internally cooled power laser for MR-guided interstitial laser-induced thermotherapy of liver lesions: initial clinical results. Radiology.

[31] Rahmathulla G, Recinos PF, Kamian K, Mohammadi AM, Ahluwalia MS, Barnett GH (2014). MRI-guided laser interstitial thermal therapy in neuro-oncology: a review of its current clinical applications. Oncology.

[32] Schwarzmaier HJ, Eickmeyer F, von Tempelhoff W, Fiedler VU, Niehoff H, Ulrich SD, Yang Q, Ulrich F (2006). MR-guided laser-induced interstitial thermotherapy of recurrent glioblastoma multiforme: preliminary results in 16 patients. Eur J Radiol.

[33] Carpentier A, Chauvet D, Reina V, Beccaria K, Leclerq D, McNichols RJ, Gowda A, Cornu P, Delattre JY (2012). MR-guided laser-induced thermal therapy (LITT) for recurrent glioblastomas. Lasers Surg Med.

[34] Mohammadi AM, Schroeder JL (2014). Laser interstitial thermal therapy in treatment of brain tumors--the NeuroBlate system. Expert Rev Med Devices.

[35] Sapareto SA, Dewey WC (1984). Thermal dose determination in cancer therapy. Int J Radiat Oncol Biol Phys.

[36] Schober R, Bettag M, Sabel M, Ulrich F, Hessel S (1993). Fine structure of zonal changes in experimental Nd:YAG laser-induced interstitial hyperthermia. Lasers Surg Med.

[37] Tracz RA, Wyman DR, Little PB, Towner RA, Stewart WA, Schatz SW, Pennock PW, Wilson BC (1992). Magnetic resonance imaging of interstitial laser photocoagulation in brain. Lasers Surg Med.

[38] Schulze PC, Vitzthum HE, Goldammer A, Schneider JP, Schober R (2004). Laser-induced thermotherapy of neoplastic lesions in the brain--underlying tissue alterations, MRI-monitoring and clinical applicability. Acta Neurochir.

[39] Kahn T, Bettag M, Ulrich F, Schwarzmaier HJ, Schober R, Furst G, Modder U (1994). MRI-guided laser-induced interstitial thermotherapy of cerebral neoplasms. J Comput Assist Tomogr.

[40] Schwabe B, Kahn T, Harth T, Ulrich F, Schwarzmaier HJ (1997). Laser-induced thermal lesions in the human brain: short- and long-term appearance on MRI. J Comput Assist Tomogr.

[41] Tracz RA, Wyman DR, Little PB, Towner RA, Stewart WA, Schatz SW, Wilson BC, Pennock PW, Janzen EG (1993). Comparison of magnetic resonance images and the histopathological findings of lesions induced by interstitial laser photocoagulation in the brain. Lasers Surg Med.

[42] Archavlis Eleftherios, Tselis Nikolaos, Birn Gerhard, Ulrich Peter, Baltas Dimos, Zamboglou Nikolaos (2013). Survival analysis of HDR brachytherapy versus reoperation versus temozolomide alone: a retrospective cohort analysis of recurrent glioblastoma multiforme. BMJ Open.

[43] Taal W, Oosterkamp HM, Walenkamp AM, Dubbink HJ, Beerepoot LV, Hanse MC, Buter J, Honkoop AH, Boerman D, de Vos FY (2014). Single-agent bevacizumab or lomustine versus a combination of bevacizumab plus lomustine in patients with recurrent glioblastoma (BELOB trial): a randomised controlled phase 2 trial. Lancet Oncol.

[44] Carpentier A, McNichols RJ, Stafford RJ, Guichard JP, Reizine D, Delaloge S, Vicaut E, Payen D, Gowda A, George B (2011). Laser thermal therapy: real-time MRI-guided and computer-controlled procedures for metastatic brain tumors. Lasers Surg Med.

[45] Carpentier A, McNichols RJ, Stafford RJ, Itzcovitz J, Guichard JP, Reizine D, Delaloge S, Vicaut E, Payen D, Gowda A (2008). Real-time magnetic resonance-guided laser thermal therapy for focal metastatic brain tumors. Neurosurgery.

[46] Rao MS, Hargreaves EL, Khan AJ, Haffty BG, Danish SF (2014). Magnetic resonance-guided laser ablation improves local control for postradiosurgery recurrence and/or radiation necrosis. Neurosurgery.

[47] Vadivelu Sudhakar, Schulder Michael (2013). MRI-Guided Interstitial Laser Therapy of Brain Tumors. Intraoperative Imaging and Image-Guided Therapy.

[48] Jo KI, Im YH, Kong DS, Seol HJ, Nam DH, Lee JI (2013). Gamma knife radiosurgery for brain metastases from breast cancer. J Korean Neurosurg Soc.

[49] Parlak C, Mertsoylu H, Guler OC, Onal C, Topkan E (2014). Definitive chemoradiation therapy following surgical resection or radiosurgery plus whole-brain radiation therapy in non-small cell lung cancer patients with synchronous solitary brain metastasis: a curative approach. Int J Radiat Oncol Biol Phys.

[50] Smith TR, Lall RR, Lall RR, Abecassis IJ, Arnaout OM, Marymont MH, Swanson KR, Chandler JP (2014). Survival after surgery and stereotactic radiosurgery for patients with multiple intracranial metastases: results of a single-center retrospective study. J Neurosurg.

[51] Kumar AJ, Leeds NE, Fuller GN, Van Tassel P, Maor MH, Sawaya RE, Levin VA (2000). Malignant gliomas: MR imaging spectrum of radiation therapy- and chemotherapy-induced necrosis of the brain after treatment. Radiology.

[52] Lampert PW, Davis RL (1964). Delayed effects of radiation on the human central nervous system; “early” and “late” delayed reactions. Neurology.

[53] Mullins ME, Barest GD, Schaefer PW, Hochberg FH, Gonzalez RG, Lev MH (2005). Radiation necrosis versus glioma recurrence: conventional MR imaging clues to diagnosis. AJNR Am J Neuroradiol.

[54] Ruben JD, Dally M, Bailey M, Smith R, McLean CA, Fedele P (2006). Cerebral radiation necrosis: incidence, outcomes, and risk factors with emphasis on radiation parameters and chemotherapy. Int J Radiat Oncol Biol Phys.

[55] Nonoguchi N, Miyatake S, Fukumoto M, Furuse M, Hiramatsu R, Kawabata S, Kuroiwa T, Tsuji M, Fukumoto M, Ono K (2011). The distribution of vascular endothelial growth factor-producing cells in clinical radiation necrosis of the brain: pathological consideration of their potential roles. J Neuro-Oncol.

[56] McPherson CM, Warnick RE (2004). Results of contemporary surgical management of radiation necrosis using frameless stereotaxis and intraoperative magnetic resonance imaging. J Neuro-Oncol.

[57] Rahmathulla G, Recinos PF, Valerio JE, Chao S, Barnett GH (2012). Laser interstitial thermal therapy for focal cerebral radiation necrosis: a case report and literature review. Stereotact Funct Neurosurg.

[58] Torres-Reveron J, Tomasiewicz HC, Shetty A, Amankulor NM, Chiang VL (2013). Stereotactic laser induced thermotherapy (LITT): a novel treatment for brain lesions regrowing after radiosurgery. J Neuro-Oncol.

[59] Fabiano AJ, Alberico RA (2014). Laser-interstitial thermal therapy for refractory cerebral edema from post-radiosurgery metastasis. World Neurosurg.

[60] DRG B, Oliva IC, Loree JM, Fuentes DT, Stafford RJ, Beechar VB, Weinberg JS, Shah K, Kumar VA, Prabhu SS (2019). Predictors of local control of brain metastasis treated with laser interstitial thermal therapy.

[61] Beechar VB, Prabhu SS, Bastos D, Weinberg JS, Stafford RJ, Fuentes D, Hess KR, Rao G (2018). Volumetric response of progressing post-SRS lesions treated with laser interstitial thermal therapy. J Neuro-Oncol.

[62] Josephson CB, Dykeman J, Fiest KM, Liu X, Sadler RM, Jette N, Wiebe S (2013). Systematic review and meta-analysis of standard vs selective temporal lobe epilepsy surgery. Neurology.

[63] Gross RE, Willie JT, Drane DL (2016). The role of stereotactic laser amygdalohippocampotomy in mesial temporal lobe epilepsy. Neurosurg Clin N Am.

[64] Drane DL, Loring DW, Voets NL, Price M, Ojemann JG, Willie JT, Saindane AM, Phatak V, Ivanisevic M, Millis S (2015). Better object recognition and naming outcome with MRI-guided stereotactic laser amygdalohippocampotomy for temporal lobe epilepsy. Epilepsia.

[65] Kang JY, Wu C, Tracy J, Lorenzo M, Evans J, Nei M, Skidmore C, Mintzer S, Sharan AD, Sperling MR (2016). Laser interstitial thermal therapy for medically intractable mesial temporal lobe epilepsy. Epilepsia.

[66] Waseem H, Osborn KE, Schoenberg MR, Kelley V, Bozorg A, Cabello D, Benbadis SR, Vale FL (2015). Laser ablation therapy: an alternative treatment for medically resistant mesial temporal lobe epilepsy after age 50. Epilepsy Behav.

[67] Tiwari P, Danish S, Madabhushi A (2014). Identifying MRI markers to evaluate early treatment related changes post laser ablation for cancer pain management. Proc SPIE.

[68] Patel Purvee, Patel Nitesh V., Danish Shabbar F. (2016). Intracranial MR-guided laser-induced thermal therapy: single-center experience with the Visualase thermal therapy system. Journal of Neurosurgery.

[69] Hirsch LR, Gobin AM, Lowery AR, Tam F, Drezek RA, Halas NJ, West JL (2006). Metal nanoshells. Ann Biomed Eng.

[70] Hirsch LR, Stafford RJ, Bankson JA, Sershen SR, Rivera B, Price RE, Hazle JD, Halas NJ, West JL (2003). Nanoshell-mediated near-infrared thermal therapy of tumors under magnetic resonance guidance. Proc Natl Acad Sci U S A.

[71] Weissleder R (2001). A clearer vision for in vivo imaging. Nat Biotechnol.

[72] Maeda H, Fang J, Inutsuka T, Kitamoto Y (2003). Vascular permeability enhancement in solid tumor: various factors, mechanisms involved and its implications. Int Immunopharmacol.

[73] Baek SK, Makkouk AR, Krasieva T, Sun CH, Madsen SJ, Hirschberg H (2011). Photothermal treatment of glioma; an in vitro study of macrophage-mediated delivery of gold nanoshells. J Neuro-Oncol.

[74] Madsen SJ, Christie C, Hong SJ, Trinidad A, Peng Q, Uzal FA, Hirschberg H (2015). Nanoparticle-loaded macrophage-mediated photothermal therapy: potential for glioma treatment. Lasers Med Sci.

[75] Wang L, Yuan Y, Lin S, Huang J, Dai J, Jiang Q, Cheng D, Shuai X (2016). Photothermo-chemotherapy of cancer employing drug leakage-free gold nanoshells. Biomaterials.

